# Effects of weaning on regulators of volatile fatty acid absorption and intracellular pH in Holstein calves

**DOI:** 10.3168/jdsc.2021-0088

**Published:** 2021-09-13

**Authors:** Rebecca L. Hiltz, Dana E. McCurdy, Steven Moreland, Keith Klanderman, Anne H. Laarman

**Affiliations:** 1 Department of Animal and Veterinary Science, University of Idaho, Moscow 83844-2330; 2 Adisseo Inc., Hampshire, IL 60140

## Abstract

This study examined the effects of changes in rumen fermentation during the weaning transition on abundance of transporters involved in volatile fatty acid (VFA) absorption or intracellular pH homeostasis. Holstein bull calves (n = 27) were assigned to 1 of 3 treatment groups in a randomized, complete block design: 2 preweaning groups [animals fed milk only (PRE-M) or milk, calf starter, and hay (PRE-S)] and 1 postweaning group (animals fed milk, starter, and hay with a 2-wk weaning transition; POST-S). Calves were euthanized at 42 d of age (PRE-M and PRE-S) or at 63 d of age (POST-S), and rumen epithelium and rumen fluid samples were collected. Rumen fluid was analyzed for VFA concentration, and rumen epithelium was analyzed for the abundance of VFA transporter monocarboxylate transporter isoform 1 (MCT1) and the intracellular pH regulators sodium bicarbonate co-transporter 1 (NBC1) and sodium-proton exchanger 3 (NHE3) protein. Preweaning, total VFA concentrations tended to increase and NBC1 abundance increased with starter intake. Between pre- and postweaning, total VFA concentrations increased but NHE3 protein abundance decreased. In calves, rumen epithelial development during the weaning transition appears to show more pronounced changes in intracellular pH homeostasis than in VFA transport capacity.

## Highlights

• Calves may experience dysregulation of intracellular pH through weaning.
• VFA concentration changes in the rumen do not equate to VFA transporter abundance.
• The calf rumen can handle large amounts of fermentable feed without negative effects.
• Cell size and relative cell circumference change as the rumen is developing.

## Graphical Abstract

**Figure:**
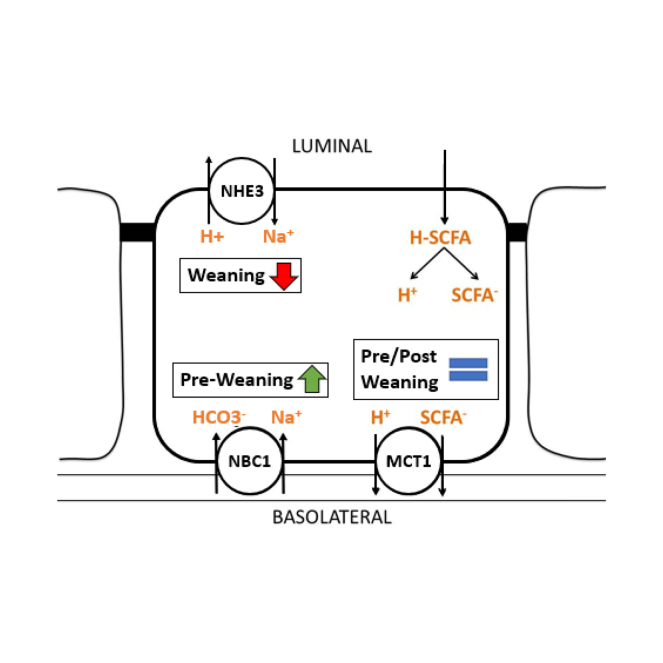
**Summary:** In calves, changes in intracellular pH regulation dynamics and volatile fatty acid (VFA) transporter abundance through the weaning transition have not previously been described. The proton exporter NHE3 decreases in abundance between the preweaning and postweaning phase. Preweaning, the bicarbonate importer NBC1 increases in abundance in calves fed milk only versus those fed milk and solid feed. With solid feed consumption, no changes in abundance for the VFA transporter MCT1 were observed preweaning or postweaning. Some short-chain fatty acids (SCFA) are still imported through passive diffusion.

Rumen development is imperative to a successful weaning transition in calves. At birth, the gastrointestinal tract of neonatal calves cannot rely on solid feed digestion alone (Górka et al., 2018), which makes anatomical and physiological development during the first weeks of life a necessity. Increased anatomical and physiological development of the rumen is driven by solid feed intake and its subsequent fermentation (Górka et al., 2018). Through solid feed fermentation, rumen VFA production increases and so does the need to transport those VFA across the rumen epithelium.

Transport of VFA across the rumen epithelium uses passive diffusion routes (Schurmann et al., 2014) and facilitated transport through membrane-bound transporters. Of the membrane-bound transporters, monocarboxylate transporter isoform 1 (**MCT1**) plays the largest role in determining VFA transport rates (Kirat et al., 2006), and it is responsible for transporter-mediated changes in VFA flux in Holstein steers (Laarman et al., 2016). Before weaning, calf starter intake increases mRNA abundance of MCT1 (Laarman et al., 2012) but not protein abundance (Yohe et al., 2019). Currently, it is unclear how VFA transporter abundance is affected by the weaning transition.

One of the comparatively unexplored facilitators of VFA transport capacity development is regulation of rumen pH and rumen epithelial intracellular pH (**pH_i_**). Increased ruminal VFA production can lower rumen pH and pH_i_ (Connor et al., 2010), and the combination of low pH and high VFA compromises barrier integrity (Meissner et al., 2017), highlighting the importance of pH_i_ homeostasis. Regulation of pH_i_ is required for cellular survival, as extreme acidification of the cell can induce apoptosis (Humer et al., 2018). Maintaining pH_i_ homeostasis involves several membrane-bound proton and bicarbonate transporters (Connor et al., 2010); among these, sodium proton exchanger 3 (**NHE3**) and sodium bicarbonate cotransporter 1 (**NBC1**) have been found to be responsive to dietary changes in adult cattle (Laarman et al., 2013a,b) and other ruminant species pre- or postweaning (Metzler-Zebeli et al., 2013; Liu et al., 2016). In a preweaning study, *NHE3* gene expression was downregulated in calves consuming calf starter (Laarman et al., 2012), but its expression levels during the weaning transition or at the protein level remain unclear. In all, pH_i_-associated transporters appear to be dynamic in expression, especially in response to dietary stimuli. Whether changes in pH_i_ regulation capacity are part of rumen epithelial development is unclear.

The objectives of this study were to examine the effect of calf starter intake during the weaning transition on rumen epithelial abundance of VFA transporter (MCT1) and regulators of intracellular pH (NHE3 and NBC1) and to study the relationship between transporter abundance and rumen fermentation profile. We hypothesized that the increased fermentation due to calf starter intake would increase abundance of VFA transport proteins and pH_i_ regulatory transporters as a compensatory response.

All animal procedures were approved by the University of Idaho Institutional Animal Care and Use Committee (AUP # 2016–32). Twenty-seven Holstein bull calves were obtained from a single commercial farm at 5 d (n = 9), 10 d (n = 9), or 16 d (n = 9) of life. All calves were fed 4 L of pooled maternal colostrum at birth, and were transported to the Palouse Research, Extension, and Education Center (Moscow, ID) in 2 groups 2 wk apart in April 2017. Upon arrival, all calves were treated with ampicillin (0.90 mg/kg of BW) at the recommendation of the attending veterinarian. From arrival at the experimental farm until being enrolled on the study at 16 d of age, calves were fed up to 1,200 g/d of milk replacer (28% CP, 18% fat; Calva Advantage, Calva Products LLC) and did not have access to solid feed. All calves were housed individually, bedded on sand, and had access to water ad libitum.

At 16 d of age, calves were enrolled in the study, blocked by age at arrival and BW at enrollment, and then allocated to 1 of 3 treatment groups, with 9 animals per treatment. Using 9 animals per treatment allowed us to detect, with 80% power, a difference of 20% between treatment means for all variables when the coefficient of variation within treatments was 15% (Berndtson, 1991). Preweaning treatments included calves fed 1,200 g/d of milk replacer only (**PRE-M**; n = 9) or 1,200 g/d of milk replacer with access to texturized calf starter (22.1% CP, 36.5% starch; Ampli-Calf Starter 20, Land O' Lakes LLC) and chopped alfalfa hay ad libitum (19.8% CP, 42.2% NDF; **PRE-S**; n = 9). A postweaning treatment (**POST-S**) included calves fed as fed as in PRE-S but going through a weaning transition, where milk replacer was reduced to 900 g/d at 42 d, further reduced to 600 g/d at 49 d, and removed completely at 56 d.

Throughout the study, calves that showed reduced milk intake, dehydration, or scours were given electrolytes (Re-Sorb, Zoetis Services LLC) at their next feeding via a bottle in addition to milk replacer feeding. All calves were fed milk replacer (28% CP, 18% fat; Calva Advantage, Calva Products LLC) at 1,200 g/d mixed with 8 L of warm water, divided into 2 feedings at 0630 and 1700 h until weaning (only POST-S calves went through a weaning transition). Milk refusals over 400 g/d were fed through an esophageal tube. In the PRE-S and POST-S groups, calves were fed starter (texturized) daily at 0700 h. Throughout the study, calf starter and hay intake were recorded daily and BW were recorded weekly.

At 35 d of age, calves in the PRE-M and PRE-S groups were dosed with a ruminal pH logger (Dascor Inc.) that recorded rumen pH continuously every 2 min for 7 d. At 42 d of age, before weaning, PRE-M and PRE-S groups were euthanized via captive bolt and exsanguination; POST-S calves began the weaning transition. At 56 d of age, after weaning, POST-S calves were dosed with a ruminal pH logger (Dascor Inc.) that recorded rumen pH continuously every 2 min for 7 d. At 63 d of age, POST-S calves were euthanized via captive bolt and exsanguination.

After euthanasia, rumen epithelium tissue and rumen fluid were collected from the ventral sac of the rumen and the rumen pH logger was retrieved. Tissue samples were rinsed with PBS (Fisher Scientific) and stored in 4% formaldehyde (Fisher Scientific) for 24 h. After 24 h, the samples were transferred to 70% ethanol until they were processed and encased in paraffin. Then, 5-µm-thick sections were mounted on slides (Washington Animal Disease Diagnostics Laboratory, Pullman, WA) for immunofluorescence analysis of membrane-bound transporters (Laarman et al., 2016). Rumen fluid samples were collected, strained through 4 layers of cheesecloth, and snap-frozen in liquid nitrogen for later VFA analysis.

Rumen pH was analyzed for mean pH and duration of ruminal pH <5.8, herein defined as the threshold for SARA. For VFA analysis, rumen fluid was acidified with 25% metaphosphoric acid and centrifuged at 24,750 × *g* for 20 min, and the supernatant was frozen at −20°C for 12 h. The supernatant was then thawed and centrifuged at 13,000 × *g* for 10 min, after which it was analyzed on an Agilent 6890 series gas chromatograph with a 7673 series injector (Agilent Technologies), with a DB-FFAP column using hexane and acetone solvents and 2-ethylbutyric acid 99%, as previously described (Hall et al., 2018).

Rumen tissue sections were blinded before staining for immunofluorescence, as done previously (Laarman et al., 2013a). Slides were deparaffinized and rehydrated with xylene, 100% ethanol, and 70% ethanol. Antigen retrieval was done using a 3% sodium citrate solution for 15 min at 95°C. The tissue was blocked and permeabilized with 10% goat serum and 0.3% Triton-X100 blocking buffer for 30 min at 95°C. Tissue was incubated at room temperature for 90 min with primary polyclonal antibodies (Novus Biologicals) for MCT1 (1:200 dilution; rabbit anti-human; catalog no. NBP1-59656), NHE3 (1:100 dilution; rabbit anti-human; catalog no. NBP1-82574), or NBC1 (1:200 dilution; rabbit anti-human; catalog no. NBP2-32020); antibody dilutions were chosen based on minimizing both image oversaturation and nonspecific binding. After triple rinsing with PBS, a fluorescent secondary antibody (1:200 dilution, goat anti-rabbit; Invitrogen, catalog no. 35552) was applied and tissue slides were incubated in the dark for 40 min. Slides were rinsed again 5 times with PBS, and a coverslip was placed using a mounting medium that contained 4′,6-diamidino-2-phenylindole (DAPI) nuclear stain (Prolong Gold Anti-Fade, Cell Signaling Technologies). A negative control without any primary antibody was stained under the specific conditions for each of the 3 antibodies tested. Stained slides were stored at −20°C until analysis.

Immunofluorescence was quantified using a confocal spinning disk microscope (Nikon TiE inverted microscope; Yokogawa X1 Spinning Disk). Settings were adjusted to minimize oversaturation and kept consistent for all samples within each antibody. Images of individual papillae were taken for each slide at 60× magnification; 3 different papillae were imaged per slide, with 3 images taken of each papilla. Quantification of transporter abundance was achieved by tracing around the perimeter of randomly selected individual cells located in the stratum basale and stratum spinosum and subsequent quantification using ImageJ software (Gavet and Pines, 2010). The measurement of each cell perimeter was defined as “relative cell circumference,” and the area within the cell perimeter was the “cell area.” Five cells were quantified per image (Laarman et al., 2016). If whole-cell signal coefficient of variation (CV) values among technical replicates exceeded 10%, an additional 5 cells per image were quantified, resulting in a range of 45 to 90 cells quantified per calf. A section of the background of each image was used to calculate corrected whole-cell signal values using the following formula (Gavet and Pines, 2010):

*WCS* = *ID_cell_* – (*A_cell_* × *M_background_*),

where *WCS* = whole-cell signal, *ID_cell_* = integrated cell density, *A_cell_* = cell surface area, and *M_background_* = mean background signal. Fluorescence was quantified using ImageJ software.

Data were analyzed using the MIXED procedure of SAS (SAS 9.4; SAS Institute Inc.) according to the following model:

*Y* = *μ* + *t_i_* + *ε_ij_*,

where *Y* is the response, *μ* is the overall mean, *t_i_* is the treatment effect, and *ε_ij_* is the residual error. Calf was included as a random effect. Relative cell circumference was used as a covariate for transporter abundance analysis. Significance was declared at *P* ≤ 0.05 and tendencies were declared at 0.05 < *P* ≤ 0.10. Numbers displayed are least squares means ± standard error of the mean, unless otherwise indicated. Production factors for this experiment (feed intake and growth) were previously described in a companion paper (McCurdy et al., 2019).

Total VFA concentrations tended to be lower for PRE-M than for PRE-S, but mean rumen pH did not differ nor did duration of SARA (Table 1). Calf starter intake and total VFA concentration were greater for POST-S calves than for PRE-S calves. Mean rumen pH and duration of SARA did not differ between treatments. Despite a 2,000 g/d increase in starter intake, a 600 g/d increase in hay intake, and an increase of over 100 m*M* in total VFA concentration between PRE-S and POST-S, mean pH and duration of SARA were unaffected.

**Table 1:** Volatile fatty acid concentration, calf starter intake, and pH parameters (LSM ± SEM) for Holstein calves during the transition from pre- to postweaning^1^, ^2^

Parameter	PRE-M	PRE-S	POST-S	*P*-value	
				PRE-M vs. PRE-S	PRE-S vs. POST-S
No. of calves	9	9	9		
Total VFA, m*M*	11.9 ± 11.8	35.6 ± 11.4	154.4 ± 11.8	0.08	<0.01
Starter intake, g/d	NA^3^	132 ± 165	2,247 ± 171	NA	<0.01
Mean pH	6.17 ± 0.21	6.25 ± 0.22	6.40 ± 0.22	0.78	0.66
Duration of pH <5.8, min/d	485 ± 188	280 ± 178	209 ± 201	0.44	0.79

1 Calves were either fed milk only (PRE-M) or were fed milk and starter and harvested preweaning (PRE-S) or 1 wk postweaning (POST-S).

2 Data from McCurdy et al. (2019).

3 Not applicable.

Abundances of epithelial cell transporters were affected by treatment. Before weaning, MCT1 and NHE3 abundance did not differ between PRE-M and PRE-S treatments (Figure 1), and the abundance of NBC1 was greater for PRE-S than for PRE-M. Relative papillae epithelial cell circumference did not differ between PRE-M and PRE-S (Figure 2). Between pre- and postweaning (PRE-S vs. POST-S treatments), MCT1 and NBC1 abundance did not differ (Figure 1), but the abundance of NHE3 was lower in POST-S calves. Also, relative papillae epithelial cell circumference was greater for POST-S calves than for PRE-S calves (Figure 2).

**Figure 1:**
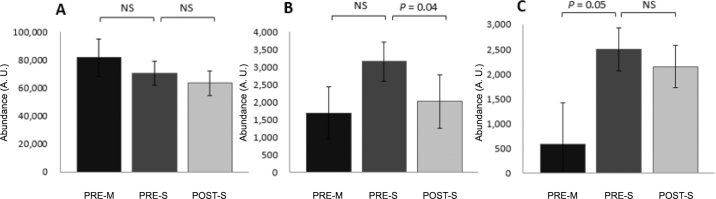
Monocarboxylate transporter isoform 1 (MCT1) abundance (A), sodium/H^+^ exchanger isoform 3 (NHE3) abundance (B), and sodium/bicarbonate co-transporter isoform 1 (NBC1) abundance (C) in Holstein calves in planned comparisons of preweaning calves (PRE-M vs. PRE-S, fed milk only and milk, starter, and hay, respectively; left bar) and weaning calves (PRE-S vs. POST-S, both fed milk, starter, and hay; right bar). No differences in MCT1 abundance were observed during preweaning or weaning (A). The relative abundance of NHE3 decreased during weaning (B; *P* = 0.04), and the relative abundance of NBC1 increased preweaning (C; *P* = 0.05). A.U. = arbitrary units. Numbers displayed are least squares means ± standard error of the mean.

**Figure 2:**
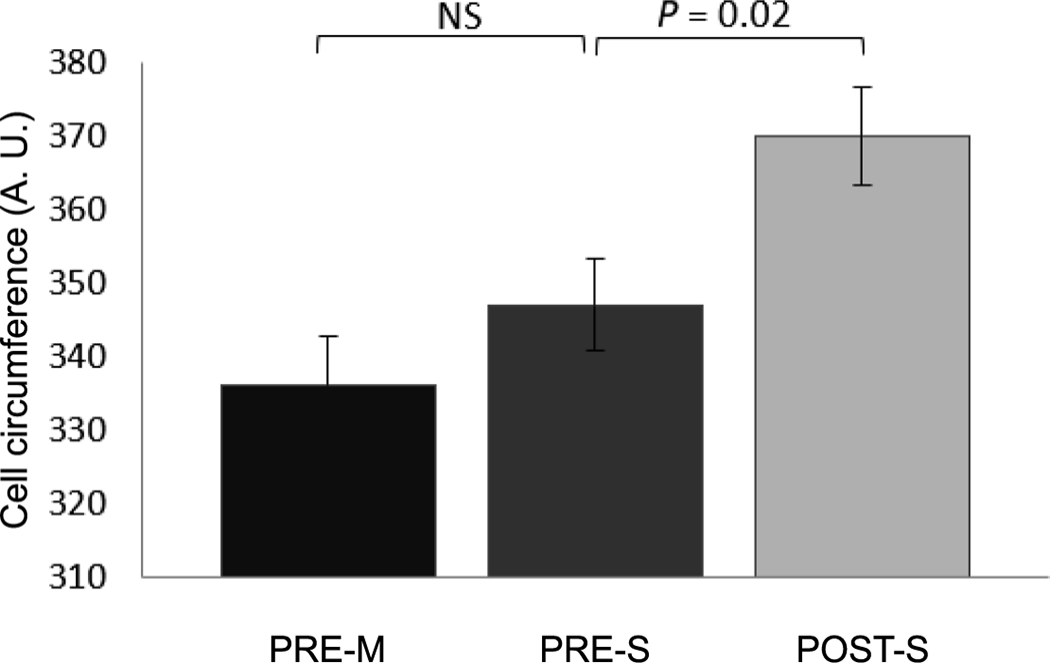
Relative rumen epithelial cell circumference for Holstein calves in planned comparisons of preweaning calves (PRE-M vs. PRE-S, fed milk only and milk, starter, and hay, respectively) and weaning calves (PRE-S vs. POST-S, both fed milk, starter, and hay). Relative cell circumference increased during weaning (*P* = 0.02). A.U. = arbitrary units. Numbers displayed are least squares means ± standard error of the mean.

The rumen fermentation dynamics in this study agree with those in other studies, suggesting that rumen pH in young calves may be affected by age more than by diet (Liu et al., 2016). In adult cows, an increase in concentrate feeding similar to this study would likely cause ruminal acidosis and a decrease in rumen pH; why such an increase in starter is tolerated in the developing calf is unknown. Although hay consumption increased between PRE-S and POST-S, concentrate consumption was more than 3 times hay consumption. The vastly different response to concentrate feeding in calves suggests that differences in buffering supply and metabolic adaptation of the rumen epithelium may be at play.

In the rumen epithelium, MCT1 is the main method of VFA export from the epithelial cytosol (Graham et al., 2007). Despite differences in rumen fermentation in the current study, MCT1 did not respond in protein abundance. There appears to be an age-related increase in MCT1 for the first 4 wk of life (Flaga et al., 2015), but the age-based trend was not found in our results. When comparing milk-only and milk and starter groups before weaning, MCT1 abundance increases at the mRNA level (Laarman et al., 2012) but is unaffected at the protein level (Yohe et al., 2019). In the current study, MCT1 was unchanged not just before weaning but also through the weaning transition. There are other factors to consider; for example, MCT1 is only one of several VFA transporters: MCT3 and MCT4 were not measured in the current study but may play a role in VFA transport (Kirat et al., 2006). Yohe et al. (2019) measured MCT4 in the rumen but found no difference between calves fed milk only and those fed milk and starter. In addition, the current study fed hay, whereas that of Yohe et al. (2019) did not. Despite the possible role of transporters, VFA clearance in the preweaning rumen is not affected by calf starter intake (Yohe et al., 2019). Together, our results and those of Yohe et al. (2019) suggest that VFA absorptive capacity is not affected by diet before weaning.

Before weaning, metabolic adaptations appear to be more geared toward pH_i_ homeostasis and barrier integrity. Before weaning, NBC1 abundance increased as a result of calf starter and hay intake. This may be due to additional bicarbonate influx via NBC1 in an attempt to regulate pH_i_ (Müller et al., 2000). Increasing the diet fermentability, which occurs around weaning, decreases pH_i_ homeostatic capacity (Laarman et al., 2015) and increases passive VFA absorption (Schurmann et al., 2014) in mature ruminants.

Between pre- and postweaning, there was a decrease in NHE3 abundance and no change in NBC1 abundance for PRE-S compared with POST-S calves. Decreased NHE3 abundance could be a factor of age or an acidifying response of the epithelium during the weaning transition; it is also possible that other transporters that were not measured, such as NHE1, were involved. A similar response is seen in decreased expression of carbonic anhydrase 2 in mature cows going through the lactation transition, suggesting that pH_i_ acidification may be taking place as part of an adaptive response to a highly fermentable diet (Laarman et al., 2015). It is important to note that the pH_i_ regulatory transporters measured here are only a few of the transporters relevant to pH_i_ homeostasis; thus, this study represents only a snapshot of the total pH_i_ regulatory response. In this study, the pH_i_ homeostatic response appears to be different before weaning than in the weaning transition; pH_i_ should be within a small range (Madshus, 1988) but may remain on opposite sides of that range for a long period. Whether the difference in response is due to age or a threshold of calf starter intake and hay is currently unclear.

At the same time as NHE3 decreased, relative cell circumference increased. This increase in cell circumference may have resulted in a dilution effect for NHE3; more cell membrane would mean less concentrated NHE3 per cell area, thus reducing the abundance measurements. The relationship between cellular growth rate and division frequency dictates the size of a cell (Tzur et al., 2009). Cells can grow without dividing and divide without growing but each change requires instructive signals (Lloyd, 2013). Cell size and rate of growth are determined by nutrient intake (Lloyd, 2013); in the current study, we observed a difference in relative papillae cell size between PRE-S and POST-S calves. It is unclear whether the cell size changes were due to the increase in available nutrients (POST-S calves had a 2,000 g/d increase in starter intake) or because of the difference in age. Like the NHE3 response, the instructive signals leading to changes in relative cell circumference are unclear, but the observed changes through weaning indicate that cell size may play a role in rumen development.

In this study, we evaluated the effect of rumen fermentation dynamics before and during weaning on VFA transport capacity and pH_i_ homeostatic capacity. Our data suggested that VFA absorption capacity is unchanged by calf starter intake. In contrast, pH_i_ homeostatic capacity showed a corrective response before weaning and an acidifying response during the weaning transition. Future research is needed to determine the role of pH_i_ homeostasis in development of the rumen epithelium.
